# Alternative splicing of the neurofibromatosis type 1 pre-mRNA is regulated by the muscleblind-like proteins and the CUG-BP and ELAV-like factors

**DOI:** 10.1186/1471-2199-13-35

**Published:** 2012-12-10

**Authors:** Victoria A Fleming, Cuiyu Geng, Andrea N Ladd, Hua Lou

**Affiliations:** 1 Department of Genetics, Case Western Reserve University, 10900 Euclid Avenue, Cleveland, OH, 44106, USA; 2 Case Comprehensive Cancer Center, Case Western Reserve University, 10900 Euclid Avenue, Cleveland, OH, 44106, USA; 3 Center for RNA Molecular Biology, School of Medicine, Case Western Reserve University, 10900 Euclid Avenue, Cleveland, OH, 44106, USA; 4 Department of Cell Biology, Lerner Research Institute, Cleveland Clinic, 9500 Euclid Avenue, Cleveland, OH, 44195, USA

**Keywords:** Muscleblind-like (MBNL) proteins, CUG-BP and ELAV-like family (CELF proteins), Alternative splicing, Neurofibromatosis type I (NF1), Splicing regulation, Complex control

## Abstract

**Background:**

Alternative splicing is often subjected to complex regulatory control that involves many protein factors and *cis*-acting RNA sequence elements. One major challenge is to identify all of the protein players and define how they control alternative expression of a particular exon in a combinatorial manner. The Muscleblind-like (MBNL) and CUG-BP and ELAV-Like family (CELF) proteins are splicing regulatory proteins, which function as antagonists in the regulation of several alternative exons. Currently only a limited number of common targets of MBNL and CELF are known that are antagonistically regulated by these two groups of proteins.

**Results:**

Recently, we identified neurofibromatosis type 1 (NF1) exon 23a as a novel target of negative regulation by CELF proteins. Here we report that MBNL family members are positive regulators of this exon. Overexpression of MBNL proteins promote exon 23a inclusion in a low MBNL-expressing cell line, and simultaneous siRNA-mediated knockdown of MBNL1 and MBNL2 family members in a high MBNL-expressing cell line promotes exon 23a skipping. Importantly, these two groups of proteins antagonize each other in regulating inclusion of exon 23a. Furthermore, we analyzed the binding sites of these proteins in the intronic sequences upstream of exon 23a by UV cross-linking assays. We show that in vitro, in addition to the previously identified preferred binding sequence UGCUGU, the MBNL proteins need the neighboring sequences for optimal binding.

**Conclusion:**

This study along with our previous work that demonstrated roles for Hu, CELF, and TIA-1 and TIAR proteins in the regulation of NF1 exon 23a establish that this exon is under tight, complex control.

## Background

Alternative splicing allows more than one protein product to be generated from a single gene by selectively including or excluding particular exons in the mature mRNA transcripts. This is a prevalent mechanism of gene regulation with as many as 94% of human genes predicted to undergo the process [1,2]. Alternative splicing is important in development, in the establishment of tissue specificity and sex differences, and in human disease etiology and progression [3-7].

Alternative splicing is a tightly regulated process involving *cis*-sequences on the RNA and protein factors that can either promote the inclusion or the skipping of a particular alternative exon in the mature mRNA. Regulatory mechanisms that govern alternative splicing have been extensively studied, and a number of splicing regulatory proteins have been identified and the *cis*-sequences to which they bind have been characterized [5,6,8-15]. More recently other means of splicing regulation have been demonstrated including chromatin remodeling and involvement of the C-terminal domain of RNA Polymerase II as a staging platform for splicing factors during coupled transcription and splicing [16-18].

Two families of well-characterized splicing regulators are the CUG-binding protein (CUG-BP) and embryonic lethal abnormal vision (ELAV) like family (CELF) and the Muscleblind-like (MBNL) proteins. CELF and MBNL proteins play important roles in the human neuromuscular disease myotonic dystrophy (DM), where their mis-regulation causes alterations in splicing patterns of their target mRNAs. In DM1, CELF protein activity is up regulated, while MBNL protein activity is lost. Interestingly, while these two groups of RNA-binding proteins are known to have distinct mRNA targets, it is also well established that they function antagonistically in the regulation of several alternative exons. The well-characterized common pre-mRNA targets that are antagonistically regulated by CELF and MBNL proteins include cardiac troponin T (cTNT) exon 5, insulin receptor (IR) exon 11, chloride channel 1 (CLCN1) exon 7a, and tau exon 6 [19-21]. Alternative splicing of these exons is mis-regulated in myotonic dystrophy. In these well-studied targets, CELF and MBNL proteins bind to distinct *cis*-elements. For example, Ho and colleagues utilized cTNT exon 5 minigene reporters in which the potential CELF or MBNL motifs were disrupted to demonstrate that the loss of one family’s binding site does not impact regulation of cTNT exon 5 by the other protein family [20]. In the case of cTNT exon 5, it has been established that MBNL proteins compete with the essential basal splicing factor U2AF^65^ for binding of the 3’ end of the cTNT intron, and when MBNL prevails it is bound to and possibly stabilizes a secondary structure that prevents U2AF^65^ binding [22]. An additional six antagonistically regulated targets were identified in a microarray analysis in the developing heart by Kalsotra and colleagues [23]. In DM1 disease, the antagonistically regulated CELF and MBNL protein splicing targets are especially adversely affected, since MBNL function is lost and CELF function is dramatically increased. For this reason, it is important to identify additional antagonistically regulated targets of these two families of regulatory proteins.

Our laboratory has identified one of the alternative exons of the neurofibromatosis type I (NF1) pre-mRNA, exon 23a, as a target of complex splicing regulation. Exon 23a is a particularly attractive exon to study because its coded amino acid sequences are located within the best-characterized domain of the NF1 protein known as the GTPase activating protein-related domain (GRD). The GRD allows the NF1 protein to mediate the conversion of active guanosine-triphosphate bound Ras (Ras-GTP) to inactive guanosine-diphosphate bound Ras (Ras-GDP) (reviewed by [24]). Interestingly, the type II isoform which includes exon 23a is ten times weaker at regulating the conversion of Ras-GTP to Ras-GDP than the type I isoform in which exon 23a is skipped [25,26]. Previously, our laboratory has shown that this exon is regulated by at least three different splicing factor protein families: CELF, Hu, and TIA-1 and TIAR [27-29].

Recently we have identified two potential MBNL binding sites, both containing UGCUGU, in the intronic region upstream of exon 23a. In this report we provide evidence to support that the MBNL family of splicing regulators act as positive regulators of NF1 exon 23a inclusion. MBNL1, 2, and 3 all promote exon 23a inclusion when over-expressed in a low MBNL protein-expressing, neuron-like cell line along with an NF1 minigene reporter. Simultaneous siRNA-mediated knockdown of endogenous MBNL1 and MBNL2 proteins in HeLa cells promotes NF1 exon 23a skipping. Our UV cross-linking assays demonstrate that recombinant MBNL1 binds to wild-type RNA oligonucleotides, but not to mutant RNA oligonucleotides in which the potential MBNL sites have been disrupted by mutation to AUAAUA. We show in cells that the relative levels of MBNL and CELF proteins govern whether or not exon 23a will be included, thus showing that CELF and MBNL proteins antagonistically regulate NF1 exon 23a. These results add NF1 exon 23a to a short list of alternative exons that are under complex control by these two families of RNA-binding proteins.

## Results

### Endogenous NF1 exon 23a inclusion patterns differ in two cell models

**Figure 1:**
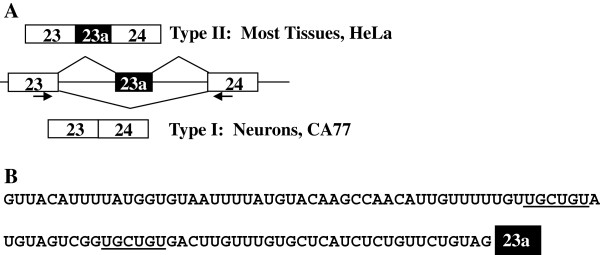
**Endogenous NF1 exon 23a inclusion patterns and UG-rich upstream intronic sequence.** (**A**) A representation of endogenous NF1 exon 23a inclusion patterns. Exon 23a is predominantly included in most tissues, while it is predominantly skipped in neurons. Arrows represent RT-PCR oligonucleotides designed to analyze NF1 exon 23a splicing patterns. (**B**) The intronic region upstream of NF1 exon 23a is UG-rich and harbors two potential Muscleblind-like (MBNL) protein binding motifs (UGCUGU) which are underlined.

Previous studies have shown that NF1 exon 23a is differentially spliced. This exon is predominantly included in most tissues, but it is mostly skipped in neurons [30,31]. To study the regulatory mechanisms by which this exon is expressed, we took advantage of two different cell model systems, HeLa cells and CA77 cells [27,29]. HeLa cells mimic the high inclusion level of NF1 exon 23a that is seen in most tissues, while CA77 cells are used to model the neuronal phenotype where NF1 exon 23a inclusion is low (Figure 1A). We use semi-quantitative, radioactive RT-PCR with primers that anneal to exons 23 and 24 to assess the inclusion pattern differences between the cells as documented previously [27]. We have shown that these cells are appropriate experimental models for examining exon 23a inclusion [27,29]. Note that according to the NF1 legacy nomenclature, exon 23 is also known as exon 23-II.

### The intronic sequence upstream of NF1 exon 23a is UG-rich and contains two potential MBNL protein binding sites

We previously demonstrated that the CELF proteins act as negative regulators of NF1 exon 23a inclusion, and that these proteins bind to the UG-rich sequences located in the intronic region upstream of exon 23a (Figure 1B and [27]). Upon closer examination of the intronic sequence upstream of exon 23a, we identified two potential binding motifs, both containing UGCUGU, for the MBNL family of splicing regulators (Figure 1B). The presence of the potential MBNL binding sites and the fact that CELF and MBNL are known to act as antagonists in the splicing regulation of several well-studied pre-mRNA targets led us to hypothesize that the MBNL proteins might also regulate NF1 exon 23a inclusion. Since the CELF proteins are confirmed negative regulators of exon 23a inclusion, we hypothesized that the MBNL family members could act as positive regulators. Given that currently all of the known common targets of MBNL and CELF proteins are regulated antagonistically by these proteins, we hypothesized that these protein families would also function antagonistically in the NF1 exon 23a system.

### Over-expression of MBNL family members in CA77 cells promotes NF1 exon 23a inclusion

**Figure 2:**
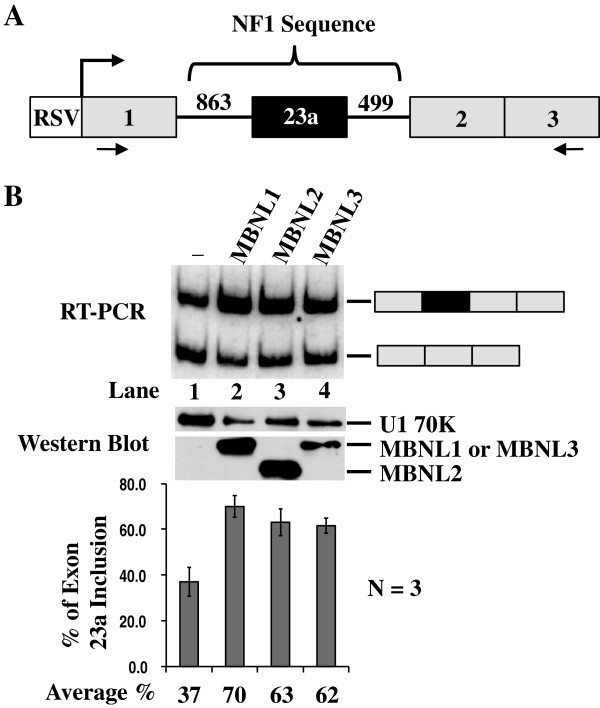
**Over-expression of the MBNL protein family members promotes NF1 exon 23a inclusion.** (**A**) Schematic representation of the HMT-NF1 863/499 minigene reporter construct. (**B**) Co-transfection of CA77 cells with the HMT-NF1 863/499 reporter (0.5 μg) and human MBNL (2 μg) protein expression plasmids. Total RNA was isolated from the transfected cells and semi-quantitative RT-PCR was performed using the primers denoted by arrows in (A). The percentage of NF1 exon 23a inclusion is displayed in the bar graph. Error bars indicate standard deviations and n=3. Total CA77 protein lysate was collected from transfected cells and Western blot analysis was performed using anti-Xpress antibody. Anti-U1 70K was utilized as a loading control.

To test the hypothesis that MBNL proteins regulate NF1 exon 23a inclusion, we performed co-transfection experiments in which individual human MBNL proteins were over-expressed along with an NF1 minigene reporter construct in CA77 cells, a low MBNL expressing cell line. The NF1 minigene reporter was created by inserting the NF1 exon 23a sequence and sequences from the flanking introns into the human metallothionine II gene (Figure 2A and [27,29]). We assessed the NF1 exon 23a inclusion pattern by semi-quantitative RT-PCR using primers that anneal to sequences in exons 1 and 3 of the human metallothionine II gene. As demonstrated by our previous work, this splicing reporter recapitulates the splicing phenotype of the endogenous NF1 pre-mRNA [27,29]. The over-expression of each of the three MBNL protein family members promoted NF1 exon 23a inclusion, indicating that they all work redundantly in this system (Figure 2B). Western blot analysis using the protein lysates from transfected CA77 cells and an anti-tag (Xpress) antibody was performed to verify that the MBNL family members were all expressed in CA77 cells (Figure 2B). As negative controls, we transfected cells with an expression plasmid for either Y-box protein or hnRNP L. Expression of neither protein had an effect on the NF1 exon 23a inclusion (data not shown). These results support our hypothesis that the MBNL protein family members are positive regulators of NF1 exon 23a inclusion.

### Knockdown of endogenous MBNL proteins promotes NF1 exon 23a skipping

**Figure 3:**
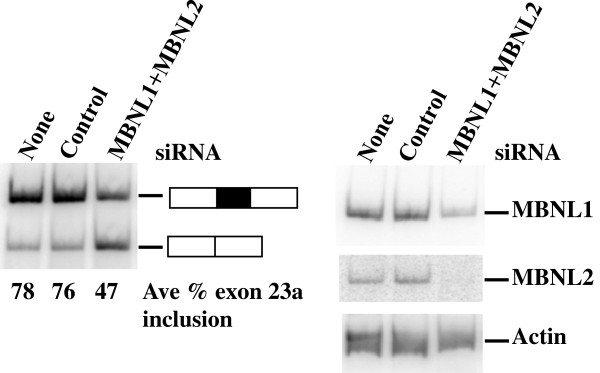
**siRNA-mediated knockdown of endogenous MBNL proteins promotes NF1 exon 23a skipping.** Co-transfection of HeLa Cells with either Control siRNA (300 pmoles) or MBNL1 (200 pmoles) plus MBNL2 (100 pmoles) siRNAs. HeLa Cells were mock transfected with no siRNA for a negative control. Total RNA was isolated from the transfected cells and semi-quantitative RT-PCR was performed using the primers designed in exons 23 and 24 (Figure 1A). The average percentage NF1 exon 23a inclusion was determined from two independent experiments. RT-PCR was performed to determine changes in MBNL1 and MBNL2 mRNA levels upon siRNA knockdown with Beta Actin as a loading control.

To establish the importance of endogenous MBNL proteins in the regulation of NF1 exon 23a inclusion, we performed a siRNA knockdown experiment in which both endogenous MBNL1 and MBNL2 proteins were disrupted in HeLa cells (Figure 3). We found that knockdown of the individual MBNL1 or MBNL2 proteins was not sufficient to change NF1 exon 23a inclusion patterns, since these proteins function redundantly in this system (data not shown). However, when the levels of both proteins were reduced endogenous NF1 exon 23a inclusion was reduced from 78% to 47% (Figure 3). We assessed MBNL1 and MBNL2 mRNA level changes, upon siRNA knockdown, using an RT-PCR assay due to difficulties with the commercially available MBNL2 antibody. As shown in Figure 3, both MBNL1 and MBNL2 mRNA levels were greatly decreased when compared with control siRNA and untreated samples. Beta Actin was used as a loading control, and there is no change in its levels upon siRNA treatment. The relatively modest change that we observed, upon siRNA knockdown of MBNL1 and MBNL2, in exon 23a inclusion was expected, since there are at least two other proteins that function as positive regulators of NF1 exon 23a in HeLa cells, TIA-1 and TIAR. In fact our results in this report are similar to our previous study in which the effects of the disruption of TIA-1 and TIAR proteins on NF1 exon 23a inclusion were assessed [29]. The loss of function experiment supports our hypothesis that the MBNL proteins are positive regulators of NF1 exon 23a inclusion.

### MBNL and CELF proteins antagonize each other in cells

**Figure 4:**
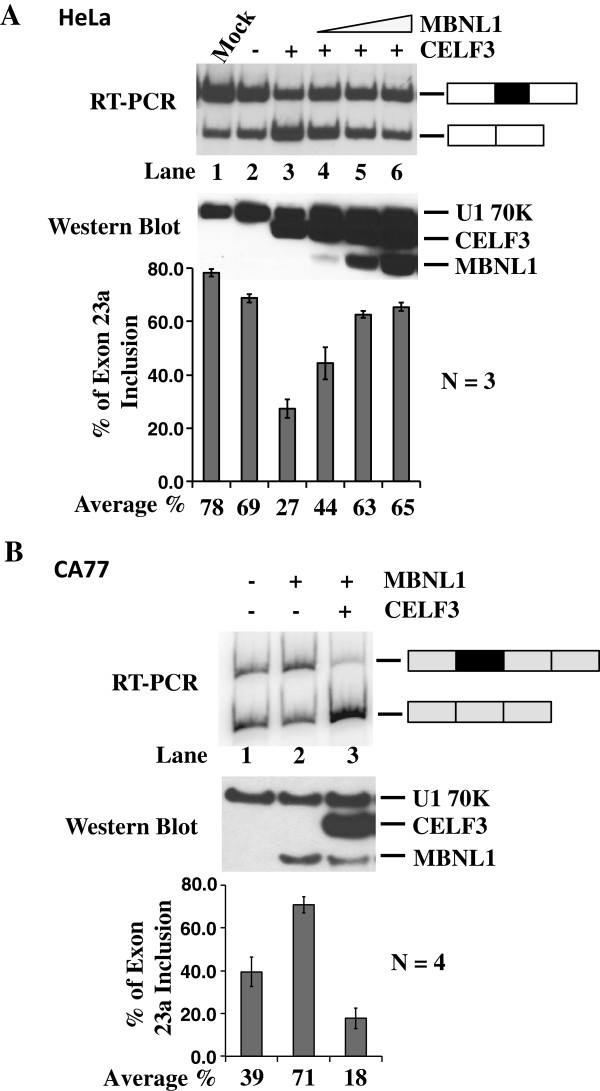
**MBNL and CELF proteins antagonize each other in cells.** (**A**) Co-transfection of HeLa cells with human CELF3 (0.5 μg) and human MBNL1 (1 μg, 2 μg, or 4 μg) protein expression plasmids. Total RNA was isolated from the transfected cells and semi-quantitative RT-PCR was performed using the primers designed in exons 23 and 24 (Figure 1A). The percentage of NF1 exon 23a inclusion is displayed in the bar graph. Error bars indicate standard deviations and n=3. Total protein was collected from the transfected HeLa cells and western blot analysis was performed using anti-Xpress antibody and anti-U1 70K as a loading control. (**B**) Co-transfection of CA77 cells with HMT-NF1 863/499 reporter (0.5 μg) and human MBNL1 (4 μg) and human CELF3 (1 μg) protein expression plasmids. Total RNA was isolated from the transfected cells and semi-quantitative RT-PCR was performed with the primers designed in exons 1 and 3 of the HMT minigene reporter. The percentage of NF1 exon 23a inclusion is displayed in the bar graph. Error bars indicate standard deviations and n=4. Total protein was isolated from the transfected CA77 cells and a western blot analysis was performed using anti-Xpress antibody and anti-U1 70K as a loading control.

Given that CELF proteins are potent negative regulators of NF1 exon 23a, we sought to understand whether the MBNL proteins could antagonize their function in cell lines. In order to test this idea, we over-expressed a constant amount of the CELF family member, CELF3, in HeLa cells and then co-expressed increasing amounts of MBNL1 protein. HeLa cells were chosen for these experiments because they are low CELF-expressing and high MBNL-expressing cells ([27] and data not shown). Since the transfection efficiency using HeLa cells is very high, we were able to assess the splicing phenotype of the endogenous NF1 pre-mRNA in these assays. Consistent with our previous studies [27], CELF3 promoted exon 23a skipping with a change from 78% inclusion in the mock-transfected cells to 27% inclusion in the CELF3 transfected cells (Figure 4A). Remarkably, with the co-expression of MBNL1, we observed a return to about 65% exon inclusion showing that MBNL1 can rescue the CELF over-expression phenotype in these cells (Figure 4A). These results suggest that overall protein levels of CELF and MBNL govern whether exon 23a will be included in HeLa cells.

Next, we wanted to determine whether a similar effect would be observed if MBNL1 and CELF3 were co-expressed in CA77 cells. CA77 were used since they express low levels of MBNL1 and MBNL2 (data not shown) and high levels of the CELF proteins [27]. CA77 cells are not efficiently transfected, so we utilized the HMT-NF1 minigene reporter as described above for these experiments. Over-expression of human MBNL1 changed NF1 exon 23a inclusion from a baseline of 39% to 71% (Figure 4B, compare lanes 1 and 2). Co-expression of human CELF3 protein with MBNL1 dramatically reduced the exon 23a inclusion level to 18%, and thus rescued the MBNL1 protein effect in CA77 cells (Figure 4B, compare lanes 2 and 3). These results demonstrate that the overall protein levels are also important in determining the expression of exon 23a in CA77 cells.

### MBNL and CELF proteins bind to the wild-type NF1 pre-mRNA but not to mutant pre-mRNAs

**Figure 5:**
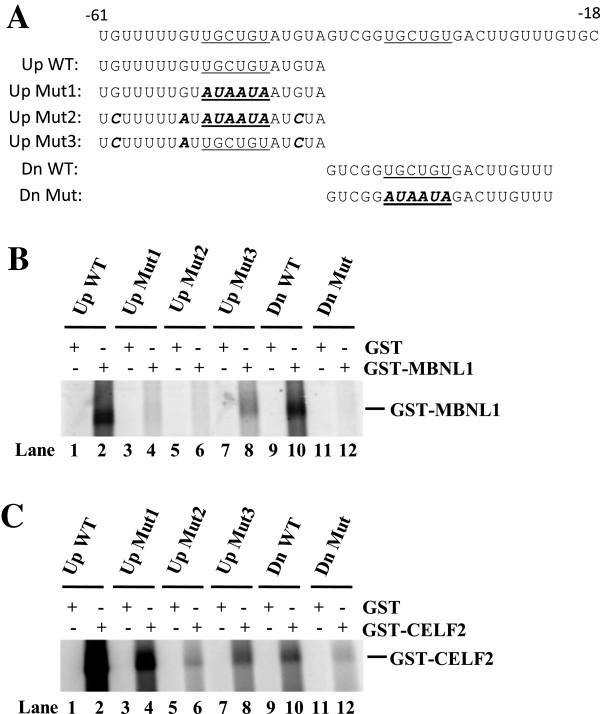
**MBNL and CELF proteins bind strongly to wild-type NF1 pre-mRNA, but not to mutant pre-mRNA.** (**A**) Sequences of RNA oligonucleotides. Potential MBNL sites are underlined. Mutations are presented in italics. The nucleotide positions relative to exon 23a are indicated. (**B**) End labeled RNA oligonucleotides were incubated with either GST as a control (750 ng) or GST-MBNL1 (750 ng), and binding was detected by UV cross-linking assays. (**C**) End labeled RNA oligonucleotides were incubated with either GST (100 ng) or GST-CELF2 (100 ng), and binding was detected by UV cross-linking assays.

Given that there are two UGCUGU-containing potential MBNL binding sites located in the intronic sequence upstream of NF1 exon 23a, and that the MBNL proteins function as positive regulators of exon 23a when over-expressed in low MBNL-expressing cells, we hypothesized that these proteins bind to the NF1 pre-mRNA. We were interested in determining whether both potential MBNL binding sites are important for binding or if one is more important than the other. We also wanted to know whether the same sites are important for CELF protein binding. To address these questions, we performed UV cross-linking assays with either wild-type or mutated RNA oligonucleotides which were end labeled with ^32^P-ATP and incubated with recombinant MBNL or CELF proteins. We designated the wild-type and mutant RNA oligonucleotides as either upstream or downstream based on the location of the potential MBNL site that they contain relative to the 3’ splice site (Figure 5A). To mutate the potential MBNL binding sites, we substituted AUAAUA for UGCUGU (upstream mutants 1 and 2 and downstream mutant in Figure 5A), based on previous in vitro studies that have shown that this sequence substitution abolishes MBNL protein binding [20]. We also disrupted potential UG dinucleotides to which CELF proteins are likely to bind in the absence or presence of UGCUGU mutation (upstream mutants 2 and 3 in Figure 5A). In these binding assays, we utilized GST protein as a negative control, and as expected it did not bind to any of the RNA sequences (Figure 5, panels B and C). GST-MBNL1 binds strongly to both the upstream and downstream wild-type RNA oligonucleotide sequences, but it does not bind to the RNA sequences in which either of the UGCUGU motifs (Figure 5B upstream mutants 1 and 2, compare lanes 2, 4, and 6, and downstream mutant, compare lanes 10 and 12) is abolished. Interestingly, we observed that GST-MBNL1 could bind to upstream mutant 3, which contains one of the proposed MBNL binding sites but lacks other potential UG dinucleotides, although this binding is greatly reduced compared with the upstream wild-type sequence (Figure 5B, compare lanes 2 and 8). This result was surprising, and it suggests that MBNL proteins might require the upstream UG sequences in addition to their UGCUGU binding site for optimal binding. Overall, these results suggest that both UGCUGU sequence motifs are important for MBNL protein binding.

We found that GST-CELF2 binds strongly to the upstream wild-type sequence, as well as to the upstream mutant 1 in which only the potential MBNL binding motif is abolished (Figure 5C, compare lanes 2 and 4). Interestingly, there is significant reduction in GST-CELF2 binding for both upstream mutants 2 and 3, with the most profound loss of binding observed with upstream mutant 2 in which both the potential MBNL binding motif and the other UG dinucleotides are changed (Figure 5C, compare lanes 2, 6, and 8). We also observed a reduction in GST-CELF2 binding for the downstream mutant, in which only the potential MBNL site was abolished (Figure 5C, compare lanes 10 and 12). These results suggest that GST-CELF2 binds optimally when there are more UG-rich sites. Taken together, the binding analysis indicates that the binding sites for MBNL and CELF proteins are somewhat overlapping in vitro.

## Discussion

### NF1 exon 23a is a novel target of MBNL protein family-mediated splicing regulation

The Muscleblind-like proteins are important, well-characterized splicing regulators [12,32]. A great deal has been learned about the MBNL proteins based on their involvement in the human disease myotonic dystrophy (DM). In order to better understand MBNL protein function, researchers have generated knockout mouse models for MBNL1 and MBNL2 [33,34]. While both of the models have some features of human DM, including aberrant splicing of known MBNL target pre-mRNAs, neither one fully recapitulates the human DM patient phenotype. Furthermore, a recent study performed using RNA isolated from the MBNL1 knockout mouse and microarray analysis identified several additional MBNL1 targets [35], but it is probable that many more targets remain to be determined.

Our studies show that all three of the MBNL protein family members function redundantly as positive regulators of NF1 exon 23a when over-expressed in CA77 cells. These findings are similar to a previous study done by Ho and colleagues, in which all of the MBNL proteins were shown to promote either exon inclusion in the case of the human insulin receptor exon 11, or exon repression in human and chicken cardiac troponin T when over-expressed in cells along with minigene reporters [20]. Although all of the family members function redundantly in the transfection experiments, MBNL3 might not have a significant role physiologically in the regulation of this alternative splicing event since its expression profile is not as extensive as that of the other two family members [36]. Furthermore, the simultaneous disruption of both of the endogenous MBNL proteins in HeLa cells by siRNA knockdown promotes NF1 exon 23a skipping which is consistent with our hypothesis that they are positive regulators and that they function redundantly in this system.

### The MBNL binding motif is located within the intronic sequence upstream of NF1 exon 23a

Using bioinformatic techniques, a recent study revealed that there is an enrichment of both MBNL and CELF binding sites surrounding developmentally regulated alternative exons, and that these sites are conserved in chicken and mouse, as well as in several other mammalian species [23]. It is possible that MBNL proteins enhance or silence the expression of their target alternative exons in a location-dependent manner. In their study, Kalsotra and coworkers found that the MBNL binding motifs located within the last 250 bases of upstream introns were significantly associated with exon skipping in MBNL-expressing cells [23,32].

Our in vitro UV cross-linking experiments have shown that recombinant MBNL proteins can bind to two UGCUGU binding motifs located upstream of exon 23a, suggesting that these binding motifs could be involved in promoting exon 23a inclusion. This result is interesting because it implies that the MBNL proteins can function as positive regulators by binding to upstream UG-rich sequences. As discussed above, MBNL binding sites located in the intronic region upstream of an alternative exon usually promote skipping of the alternative exon. For example, the MBNL binding sites for exon 5 of the cardiac troponin T (cTNT) pre-mRNA are located in the intron upstream of exon 5, and consistent with the bioinformatics data, MBNL proteins promote exon 5 skipping [20]. Also, in the case of insulin receptor exon 11 regulation, the MBNL protein binding sites are located downstream of exon 11 and MBNL proteins promote the inclusion of this exon [20]. The functional significance of the two UGCUGU motifs needs to be further investigated using mutant minigenes to confirm that these sites indeed function in the MBNL protein-mediated splicing regulation of NF1 exon 23a.

Recombinant MBNL1 protein binds strongly to the upstream-most wild-type RNA sequence, which contains one of the potential MBNL1 binding sites, but as predicted, binding was lost when either the MBNL site alone or both the MBNL and potential CELF binding sites were abolished (Figure 5B, compare lanes 2, 4, and 6). Surprisingly, the recombinant MBNL1 protein could not bind as strongly to the RNA sequence in which the potential MBNL site was intact but other UG elements were abolished (Figure 5B, compare lanes 2 and 8). This result suggests that for optimal binding the MBNL proteins require additional upstream UG sequences. In the literature, a number of MBNL binding sites have been identified in both pre-mRNA targets and in the CUG or CCUG repeats associated with myotonic dystrophy [20,37-42]. The common features of these binding sites are that they are pyrimidine-rich themselves and are usually surrounded by pyrimidine-rich sequences, and they generally feature the YGCY (where Y is a pyrimidine) motif [42]. While the two predicted MBNL binding sites upstream of NF1 exon 23a fit the profile for containing the YGCY motif (UGCUGU), the additional sequence that was abolished from upstream mutant 3 does not have this motif (Figure 5A). However, the entire region upstream of this exon is pyrimidine-rich, which is consistent with the current knowledge about other intronic regions surrounding MBNL protein-regulated alternative exons. It is interesting that switching only one residue from a pyrimidine to a purine can have such a profound effect on the binding of MBNL1 (Figure 5A, compare upstream wild-type sequence with upstream mutant 3 sequence). Perhaps this effect is so strong because the pyrimidine to purine change was made in such close proximity to the predicted MBNL binding site. It is also possible that this change could have disrupted an RNA secondary structure or some other mechanism that promotes optimal MBNL1 binding to the pre-mRNA. It has been shown that MBNL proteins can regulate some of their pre-mRNA targets by binding to the stem of RNA stem-loop structures containing their binding sites [40,41]. Using a structure prediction software program, we found that stem-loop structures are predicted to form upstream of exon 23a, and that these structures involve the two potential UGCUGU binding motifs (data not shown). Furthermore, these predicted secondary structures are conserved in humans, mice, and chickens. The role of the potential secondary structure in MBNL-mediated inclusion of NF1 exon 23a remains to be investigated.

### NF1 exon 23a is antagonistically regulated by the MBNL and CELF protein families

Our over-expression studies in CA77 and HeLa cells have demonstrated that the levels of CELF and MBNL proteins are important for determining whether NF1 exon 23a will be included or skipped, and these findings are consistent with the hypothesis that CELF and MBNL proteins act as antagonists in the regulation of this alternative splicing event. The identification of NF1 exon 23a as a new target of CELF and MBNL protein antagonistic splicing regulation is important, because NF1 has a critical role in the developing heart. It has been shown previously that mice deficient for *Nf1* die at mid-gestation due to heart development-related complications, and that there is an important mesenchymal to endothelial transition at this stage in mouse heart development for which Nf1 is important [43]. NF1 exon 23a is located within the GRD of the neurofibromin protein, and the two NF1 isoforms differ in their abilities to negatively control Ras signaling. Also this exon is under complex control by at least four groups of regulatory proteins, suggesting that its function is essential in the proper management of Ras signaling *in vivo*. Thus, it is intriguing to hypothesize that NF1 signaling could be disrupted in the hearts of DM1 patients since the inclusion of NF1 exon 23a is antagonistically regulated by the CELF and MBNL proteins.

In the known antagonistically regulated pre-mRNA targets, CELF and MBNL proteins bind to distinct binding sequences. Ho and colleagues used minigene reporters for cTNT exon 5 and insulin receptor exon 11 with either MBNL or CELF sites disrupted to demonstrate that neither protein needs the other protein’s site in order to regulate the alternative exon [20]. Our in vitro binding assays suggest that there may be some overlap in binding sequences for the MBNL and CELF proteins on the NF1 pre-mRNA. In Figure 5C, we show that recombinant CELF2 binds strongly to the upstream RNA sequence, but its binding is reduced for all three mutants (compare lanes 2, 4, 6, and 8). In addition, although binding to the downstream sequence is not as strong as to the upstream sequence, there is also a great reduction in binding to the downstream MBNL site mutant. Since the MBNL sites are UG-rich, it is not surprising that the recombinant CELF2 protein binds better when there is more of that type of sequence available. In our work, we have used two representative CELF proteins to study the antagonistic relationship between CELF and MBNL proteins. Previous studies have shown that CELF2 and CELF3 can behave differently in other systems [44,45], but the two proteins function redundantly for NF1 exon 23a [27] and therefore may be used interchangeably in our experiments.

## Conclusions

In summary, the study reported here adds NF1 exon 23a to a short list of pre-mRNAs that are antagonistically regulated by the CELF and MBNL protein families. These studies also add an additional positive regulatory factor to the list of proteins and regulatory mechanisms that control the expression of NF1 exon 23a. These findings are especially interesting because they suggest a novel mechanism by which the MBNL and CELF proteins can function antagonistically, since there may be some overlap between their binding motifs as demonstrated by our in vitro binding assays.

## Methods

### Plasmids

The human NF1 minigene reporter was previously described [27,29]. The protein expression plasmids for CELF3, MBNL1, MBNL2, MBNL3 and Y-Box protein were gifts from Dr. Tom Cooper at Baylor College of Medicine. The expression plasmid for hnRNP L was a gift from Dr. Kristen Lynch at University of Pennsylvania.

### Cell culture and cell transfections

HeLa and CA77 cells were cultured and transfected as previously described [27,29]. HeLa cells were obtained from American Type Culture Collection (Manassas, VA) and CA77 cells, a cell line derived from rat medullary thyroid carcinoma (a gift from Dr. Andrew Russo, University of Iowa, Iowa City, IA) [46,47].

### RNA and protein analysis

The procedures for the isolation of total RNA and protein and for RT-PCR were performed as previously described [27,29]. Western blot analysis to analyze MBNL1 and CELF protein expression were carried out using either 50 μg of total protein lysate from transfected HeLa cells or 100 μg of total protein lysate from transfected CA77 cells loaded onto 10% polyacrylamide gels. Proteins were transferred to polyvinylidene fluoride (PVDF) membranes at 4°C overnight at 40 Volts. Following overnight transfer, the membranes were blocked in a 5% milk/PBST solution for one hour and then blotted with Anti-Xpress antibody (Invitrogen) at a dilution of 1:2000 and Anti-U1 70K at a dilution of 1:250 as a loading control for one hour. The membranes were then washed three times for 5 min each in 1X PBST, and then subjected to blotting with Goat Anti-Mouse secondary antibody (Pierce) at a dilution of 1:1250. After three final washes in 1X PBST for 5 min each, the HeLa cell blots were incubated with Pierce Pico HRP substrate for 15 min and exposed to X-ray film. For proteins derived from CA77 cell transfections, blots were incubated for five minutes in Immobilon Western Chemiluminescent HRP substrate (Millipore), and then exposed to X-ray film.

### siRNA-mediated knockdown of MBNL1 and MBNL2

The siRNA duplexes were synthesized by Dharmacon (Thermo Scientific). We used the Dharmacon MBNL2 SMARTpool siRNA, and the target sequence of the MBNL1 siRNA is 5^′^AACACGGAAUGUAAAUUUGCA3^′^ as previously described by Ho and colleagues [20]. For a negative control, we used siRNA against human USP13, which is a deubiquitination enzyme, and its target sequence is 5^′^UGAUUGAGAUGGAGAAUAA3^′^. Co-transfections were performed in HeLa cells using a total of 300 pmoles of either control siRNA as a negative control, or 200 pmoles of MBNL1 siRNA plus 100 pmoles of MBNL2 siRNA using DharmaFECT1 (Dharmacon). RT-PCR was utilized to detect changes in endogenous levels of MBNL1 and MBNL2 mRNA upon siRNA knockdown and Beta Actin was used as a loading control. The sequences for the RT-PCR oligonucleotides are: Beta Actin (Sense: 5^′^TGGGCGACGAGGCCCAGAGCA3^′^ and Antisense: 5^′^GTCAGGTCCCGGCCAGCCAGG3^′^); MBNL1 (Sense: 5^′^ATGGCTGTTAGTGTCACACCA3^′^ and Antisense: 5^′^CATGTTCTTCTGCTGAATCAA3′); MBNL2 (Sense: 5^′^CAGGTTGAAAATGGAAGAGTAA3^′^ and Antisense: 5^′^TTGAGCCCGGGACAGTGACCGG3^′^).

### UV cross-linking assays

Recombinant GST and GST-CELF2 were prepared from bacteria using the B-PER GST fusion protein purification kit (Pierce/Thermo Scientific), and they were dialyzed into Roeder D [48]. Recombinant GST-MBNL1 was prepared as previously described [49], and then dialyzed into Roeder D. RNA oligonucleotides were synthesized commercially with a protective cap by Dharmacon/Thermo Scientific. The RNA oligonucleotides were subjected to a mild deprotection protocol prior to being end-labeled with ^32^P-ATP. UV cross-linking assays were carried out in a volume of 25 μL containing 2 mM ATP, 20 mM creatine phosphate, 0.6 mM MgCl2, 1.5% polyethylene glycol, 0.15 mM dithiothreitol, and 5 x 10^5^ cpm of ^32^P-labeled RNA, and either 100 ng of GST or GST-CELF2 or 750 ng GST or GST-MBNL1. Reaction mixtures were incubated at 30°C for 30 min, and heparin was added to a final concentration of 2 μg/μl, followed by UV irradiation (254 nm) at 4°C for 15 min. The cross-linked proteins were analyzed using sodium dodecyl sulfate-10% polyacrylamide electrophoresis gels.

## Abbreviations

cTNT: Cardiac troponin t; CELF proteins: CUG-BP and ELAV-Like family proteins; CLCN1: Chloride channel 1; ELAV: Embryonic Lethal Abnormal Vision; Ras-GTP: Guanosine-triphosphate bound Ras; Ras-GDP: Guanosine-diphosphate bound Ras; GRD: GTPase-activating protein related domain; IR: Insulin receptor; mRNA(s): Messenger ribonucleic acid(s); MBNL proteins: Muscleblind-like proteins; DM: Myotonic dystrophy; DM1: Myotonic dystrophy type I; NF1: Neurofibromatosis type I; RNA: Ribonucleic acid; TIA-1: T-cell intracellular antigen 1; TIAR: T-cell intracellular antigen 1 related protein; U2AF^65^: U2 auxiliary factor large subunit.

## Competing interests

The authors declare that they have no competing interests.

## Authors’ contributions

VF and HL conceived and designed the experiments, analyzed the data, and wrote the manuscript. VF and CG performed the experiments. AL contributed reagents and materials and provided input into the project’s direction. All authors read and approved the final manuscript.
